# Overexpression of Nicotinamide N-methyltransferase mainly covers stroma of colorectal cancer and correlates with unfavorable survival by its product 1-MNA

**DOI:** 10.7150/jca.56419

**Published:** 2021-08-26

**Authors:** Jun Yang, Qingchao Tong, Ying Zhang, Shijin Yuan, Yuzhen Gao, Ke Deng, Yanzhong Wang, Jie Lu, Xinyou Xie, Zhe Zhang, Jun Zhang

**Affiliations:** 1 Department of Clinical Laboratory, Sir Run Run Shaw Hospital, Zhejiang University School of Medicine, 3 East Qingchun Road, Hangzhou 310016, Zhejiang, People's Republic of China; 2 Key Laboratory of Biotherapy of Zhejiang Province, 3 East Qingchun Road, Hangzhou 310016, Zhejiang, People's Republic of China; 3 Ningbo Diagnostic Pathology Center, 685 North Huancheng Road, Ningbo 315010, Zhejiang, People's Republic of China; 4 Department of Pathology, Ningbo Medical Center Lihuili Hospital, 57 Xingning Road, Ningbo 315040, Zhejiang, People's Republic of China; 5 Department of colorectal surgery, Ningbo Medical Center Lihuili Hospital, 57 Xingning Road, Ningbo 315040, Zhejiang, People's Republic of China

**Keywords:** Nicotinamide N-methyltransferase, colorectal cancer, tumor stroma, survival, metastasis, 1-methyl-nicotinamide

## Abstract

**Background:** Accumulating evidence indicates that Nicotinamide N-methyltransferase (NNMT) is abnormally expressed in tumor tissues of several cancers including colorectal cancer (CRC) and associated with cancer progression. However, the distribution characteristics and the clinical value of each part of NNMT expression in CRC are still not fully understood. The purpose of this study is to determine the distribution of NNMT expression and its association with survival in CRC.

**Methods:** By using the cancer genome atlas (TCGA) and clinical proteomic tumor analysis consortium (CPTAC), we firstly analyzed the difference of gene and protein levels of NNMT between CRC and normal colorectal tissue. Then, NNMT protein expressions were detected in 18 intraepithelial neoplastic samples and 177 CRC tumor samples through immunohistochemistry in our study cohort. Furthermore, the relationship between NNMT expression and clinicopathological characteristics, overall survival (OS) and disease-free survival (DFS) of CRC patients were analyzed by Pearson χ2 test and log-rank test, respectively, in public datasets and our study cohort. Lastly, the function of NNMT and its product 1-methyl-nicotinamide (1-MNA) on migration and invasion in colorectal cancer cells was analyzed by wound healing assay and transwell assay.

**Results:** We determined that higher NNMT expression in CRC tissues than normal tissues in both gene and protein level in TCGA and CPTAC datasets (all *p* < 0.05). In addition, the strong relationships of NNMT expression with stromal cells were found in the TCGA cohort. Fortunately, our cohort could validate that the expression of NNMT in tumor stroma cell was significantly higher than that in tumor cell (*p* < 0.0001), and both of them were significantly higher than that in adjacent normal tissue (ANT) (*p* < 0.0001 and *p* < 0.0001, respectively). Furthermore, the positive NNMT expression in tumor cell and stromal cell were associated with series of unfavorable clinical characteristics, including advanced TNM stage, lymph node metastasis, distant metastasis (all *p* < 0.05). Also, higher NNMT was associated with unfavorable survival both in our study and public datasets, including TCGA and two Gene Expression Omnibus (GEO) datasets (GSE33113 and GSE17538). Moreover, the functional experiments showed that stromal cells with high NNMT expression can secret 1-MAN to promote migration and invasion of CRC cells *in vitro*.

**Conclusions:** In CRC, NNMT is overexpressed in tumor cells and stroma cells, and then mainly expressed in tumor stroma cells. Overexpression of NNMT in tumor cell and stroma cell both are associated with metastasis and unfavorable survival. Besides, stromal cells with high NNMT expression secrets 1-MAN to promote migration and invasion of CRC cells. Therefore, NNMT may be a potential prognostic indicator in CRC patients.

## Introduction

Colorectal cancer (CRC) is the third most common cause of cancer death in both men and women in the United States. During the recent 5 years, the annual CRC incidence rate was 38.7 per 100,000 persons 1. In China, the incidence of CRC will reach 20.7 per 100,000 persons in 2020, which has rapidly increased and brought substantial public health burden 2. The current treatment strategies for CRC patients present favorable therapeutic efficacy, including surgery, radiotherapy, chemotherapy, target therapy and immunotherapy, however, the mortality of CRC is still high. According to the statistic, approximately 25% of CRC patients have metastasized tumors prior to diagnosis 3 and more than one-half patients developed liver metastases during the disease process, which may be the major cause of cancer-related death in CRC 4, 5.

Nicotinamide N-methyltransferase (NNMT) is a form of vitamin B3 and a cytosolic enzyme with a molecular mass of 29 kDa, which catalyzes the N-methylation of pyridine-containing compounds using the cofactor S-5′-adenosyl-L-methionine (SAM) as the methyl group donor 6. 1-methylnicotinamide (1-MNA), an endogenous metabolite of nicotinamide (NA), is generated by NNMT 7. Accumulating evidence indicates that NNMT is highly expressed in numerous malignancies, including renal cell cancer 8, breast cancer 9, 10, non-small cell lung cancer 11, 12, bladder cancer 13, cervical cancer 14, gastric cancer 15, 16, ovarian cancer 17, 18, esophageal squamous cell carcinoma 19, hepatocellular carcinoma 20, 21 and endometrial cancer 22. In addition, NNMT is found to be positively correlated with unfavorable clinicopathological features based on previous studies, including advanced clinical stage, distant metastasis and poor prognosis. Our previous study has reported the effect of NNMT on promotion of cell cycle progression in CRC cells due to the concentration of 1-MNA 23. Recently, NNMT was found to be overexpressed in stroma of CRC 24 and cancer associated fibroblasts of gastric carcinoma 25 by immunohistochemical analysis of clinical samples. Therefore, the effect of NNMT expression in stromal cells surrounding tumor tissue on tumorigenesis and development through tumor microenvironment is worth studying.

Tumor microenvironment serves a critical role in most of tumor progression processes, which is composed of tumor cells, surrounding cancer-associated fibroblasts (CAFs), blood vessels, extracellular matrix (ECM), inflammatory cells and basement membrane and so on. Stromal CAFs are the critical components of the tumor mesenchyme and not only provide physical support for epithelial cells but also play a vital role in controlling cancer progression and metastasis by secreting of a variety of cytokines, oncogenic extracellular matrix components and signaling molecules 26-33.

Previous majority studies merely focused on the expression of NNMT in tumor cell while the investigation of tumor stroma and the relative mechanism on tumor metastasis were limited. Here, we aimed to explore the expression of NNMT on tumor stroma and the molecular mechanism between NNMT expression and tumor metastasis. Besides, we also aimed to assess the potential valuation of NNMT expression as a biomarker for prognosis of CRC patients.

In this study, we found that high NNMT expression both in tumor cell and stroma were associated with unfavorable clinical characteristics of CRC patients, including advanced TNM stage, lymph node metastasis and distant metastasis as well as poor OS. Human colon fibroblast cell line CCD-18Co with high NNMT expression secrets 1-MNA, which leads to an increased the invasion and migration capability of CRC cells.

## Materials and methods

### Public Colorectal Dataset Collection

To identify the different expression of NNMT between colorectal cancers and normal tissue, the RNA transcriptome gene expression data of NNMT were extracted from the TCGA (downloaded from the XENA, https://xenabrowser.net/), and the proteomics data of that were downloaded from the project of “CPTAC Colon Cancer Confirmatory Study” (BioProject Accession: PRJNA514017 ID: 514017, https://cptac-data-portal.georgetown.edu/study-summary/S045). In addition, we also collected the datasets from TCGA, GSE33113 and GSE17538 to estimate the relationship of NNMT with survival of colorectal patients. All data were normalized for analysis by the package of “limma”, for both GEO platform and Illumina platform. The xCell R package was used to generate the infiltration of the stromal cells by the online tool “http://xcell.ucsf.edu/”.

### Tissue samples and clinical data collection

Between May 2013 and July 2014, 106 tissue samples of CRC patients after operative resection and 18 intraepithelial neoplastic (IN) samples were collected from Ningbo Diagnostic Pathology Center, Ningbo, China. In addition, colorectal cancer tissue microarray (TMA) (DC-Col01022), including 72 cancer tissue and 8 normal tissue, were provided by Avilabio Biotechnology Ltd.,Co. (Xian, China). In 178 cancer tissue samples, one cancer tissue was excluded due to unqualified sample and remained total of 177 cases. Among them, 92 cases had corresponding ANT, and 106 cases had follow-up data that could be used for survival analysis. The clinical characteristics of CRC patients were extracted from their medical record, including age, gender, histology, TNM stage.

The inclusion criteria were as follows: (a) patients who were pathological diagnosed of colorectal cancer and the TNM stage according to the World Health Organization (WHO) classification for the tumors of digestive system 2010 version. (b) patients without anti-cancer therapies prior to surgery. The exclusion criteria were as follows: (a) the patients had other synchronous malignancies or serious systemic diseases. (b) Recurrence and metastases tumor in colon and rectum. (c) Patients does not provide signed informed consent for research. This study was approved by the Human Research Ethics Committee of Sir Run Run Shaw Hospital.

### Immunohistochemistry (IHC) analysis on paraffin tissue samples

The tissue samples were fixed in formalin and embedded with paraffin. The paraffin-embedded tissue samples were cut into 4μm-thick sections and baked at 65 °C for 2h. Briefly, the sections were deparaffinized and hydrated. The antigen was retrieved with 0.01 M citrate buffer pH 6.0 and microwave heat induction. Then, the sections were treated with 1% H_2_O_2_ for 5 mins to block the endogenous peroxidase activity. After washed with PBS, non-specific binding was blocked by normal goat serum at room temperature for 10 mins. The sections were incubated with mouse monoclonal anti-human NNMT antibody (1E7 8, dilution 1:1400) in a moist chamber for 40 mins and with biotinylated goat anti-rabbit secondary antibody for 30 minutes. Visualization was achieved with streptavidin-horseradish peroxidase and freshly prepared diaminobenzidine (DAB). Subsequently, the sections were counterstained with hematoxylin, dehydration, transparency and sealing. Finally, the images of IHC were captured by digital slide scanning system (KF-PRO-005, Ningbo Jiangfeng Biologocal Information Technology Co. Ltd.).

The expression of NNMT was scored using the semi-quantitative H-score method, which takes into account both the staining intensity and the percentage of cells at that intensity. The expression level of NNMT was evaluated by two experienced pathologists, who were blinded to clinical information. The scoring system for the classification of NNMT expression in tumor cell level was scored as 0 (no staining), 1+ (weak staining), 2+ (moderate staining), or 3+ (intense staining), we also calculated the percentage of intensity staining for each specimen. Then, the final staining score was calculated by multiplying the percentage of positive cells and the respective intensity. Therefore, the staining score had a minimum value of 0 and a maximum value of 300. The scoring system for the classification of NNMT expression in tumor stroma level in the same way. The difference between the observers was averaged. The final score was classified as negative and positive expression group in tumor cell using the cut-off value and as low and high expression group in tumor stroma using the median value.

### Cell lines and cell culture

The CRC cell line HT-29, HCT116, DLD1, SW620 and SW480 cells were purchased from the Cell Bank at Chinese Academy of Sciences (shanghai, China). Human colon fibroblast cell line (CCD-18Co) was obtained from the American Type Culture Collection. All the cancer cells were cultured in RPMI-1640 medium (Gibco, Grand Island, NY, USA). CCD-18Co was cultured in Eagle's minimal essential medium. These media were supplemented with 10% fetal bovine serum (Gibco, Long Island, NY, USA), 100 U/ml of penicillin (Sigma, St. Louis, MO, USA) and 100 ug/ml of streptomycin (Sigma, St. Louis, MO, USA). The cells were maintained at 37℃ in a humidified 5% CO_2_ incubator.

### NNMT overexpression in SW480 cells and its downregulation in HT-29 cells and CCD-18Co cells

The overexpression models of SW480 cells and the downregulation models of HT-29 cells were conducted as previously described 10. Briefly, the pcDNA3.1/NNMT or pcDNA3.1 vector was transfected into SW480 cells using Lipofectamine™ 2000 (Invitrogen Life Technologies, Carlsbad, CA, USA), and the transfected cells were cultured in complete medium containing 600 mg/L geneticin (G418; Gibco, Grand Island, NY, USA). After G418 selection for two weeks, single colonies were transferred separately to 96-well plates and allowed to proliferate, Western blot analysis as described below. One monoclonal cell strain transfected with the pcDNA3.1 vector, which is named SW480/Vector, was used as a negative control. Two monoclonal cell strains with stable NNMT overexpression, namely SW480/NNMT-1 and SW480/NNMT-2, were selected for further analysis. HT-29 and CCD-18Co cells were seeded in six-well plates and incubated for 24 h. When the cells reached 30-50% confluence, the lentivirus containing the NNMT shRNAs (NNMT shRNA-1, NNMT shRNA-2, shRNA NC, MOI = 10 for HT-29 and CCD-18Co) was added to the cell culture. Ten hours after co-culturing with the lentivirus, the supernatant was replaced with fresh medium. Forty-eight hours after infection, the transduced cells were using a BD FACS Aria II System (BD Biosciences, San Jose, CA, USA) sorted to obtain the GFP-positive cell populations, and these populations were then subjected to functional assays. The efficiency of gene silencing was detected by Western blot analyses as described below. Cells infected with shRNA NC were used as the negative control.

### Western blot analysis

Protein samples were extracted from cells using RIPA lysis buffer supplemented with a protease inhibitors cocktail. The protein lysates were separated by SDS/PAGE and transferred to Immobilon P transfer membranes (Millipore, Bedford, MA, USA) that were blocked with skim milk powder at room temperature for an hour. The blots were then incubated at 4℃ overnight with primary antibodies targeting NNMT (1E7, 1:1000) and β-actin (Cell Signaling Technology, #5125). Following incubation with the secondary antibody at room temperature for an hour, the bands were visualized using enhanced chemiluminescence detection reagents (Millipore, Billerica, MA, USA). Each assay was repeated three times.

### Wound healing assay for assessing cell migration

Wound healing assay was used to assess the migration ability of HCT116, SW480 and CCD-18Co cells. In brief, cells were seeded at a density of 8x10^6^ cells/well for 24 h and treated with 1-MNA (0, 1.0, 2.0 mM). The confluent monolayer of cells was then wounded using a p200 pipette tip. The existing media was replaced with fresh RPMI-1640. For each well, five images were captured with a microscope for each group at 0 and 48 h. All experiments were performed in triplicate.

### Transwell assay for assessing cell invasion

Transwell assay was performed using Boyden chambers (8‑µm pore filter; Corning Inc., Corning, NY, USA). The filter surfaces were precoated with Matrigel (BD Biosciences, Franklin Lakes, NJ, USA). In brief, cells were seeded at a density of 1x10^6^ cells/well for 48 h in the upper chamber in RPMI-1640 medium without FBS and RPMI-1640 medium (600 µl) with 20% FBS was plated in the lower chamber. Then, cells were treated with 1mM or 2 mM 1-MNA or H_2_O as control. After 48 h of incubation, non-invading cells were removed with cotton swabs. The invasive cells located on the lower side of the chamber were fixed in paraformaldehyde for 30 min at 37˚C and stained with 0.5% crystal violet overnight at room temperature. Stained cells were counted in five random fields using fluorescence microscopy (magnification, x40). All experiments were performed in triplicate.

### 1-MNA measurement by LC-MS/MS

The 1-MNA was detected by LC-MS/MS with AB SCIEX Triple QuadTM 4500MD mass spectrometry system. For intracellular 1-MNA, cells first were treated with methyl alcohol and the supernatant was collected by centrifugation at 10,000 rpm for 15min. Then, 250μL of 1% zinc sulfate solution with stable isotope labelled internal standard (N-MNA-d4) was added into 50μL cell supernatant or culture medium. After shaken at 400 rpm for 30 min and centrifuged at 14,000 rpm for 20 min, the supernatant sample was transferred to glass vials for LC-MS/MS. Liquid chromatography separation was performed by injecting 5μL sample into the Eclipse XDB-C18 column (4.6x150 mm, 5μm; Agilent) connected to JasperTM (SCIEX) LC system. The MS/MS detection of 1-MNA and N-MNA-d4 (internal standard) was performed in multiple reaction monitoring (MRM) mode. A series of concentration standards (1-MNA) was used for the quantification of 1-MNA in samples. The final intracellular 1-MNA concentration is quantified based on the protein of the cell.

### Statistical analysis

Numerical data were presented as mean ± standard error. The student test was used to determine the statistical significance of differences between comparison groups in vitro. The relationships between NNMT expression and clinicopathological attributes were analyzed using Pearson's χ2 test. Heat map was plotted by the “ComplexHeatmap” R package. The best truncation of NNMT in the public cohort was determined by the surviminer R package. Survival curves were plotted by the Kaplan-Meier method with log-rank test. *p* < 0.05 was considered statistically significant. All statistical analyses were carried out using SPSS 21.0, R 4.0 and Graphpad prism7.0.

## Results

### NNMT is highly expressed in tumor cell and stroma in CRC

**Figure 1:**
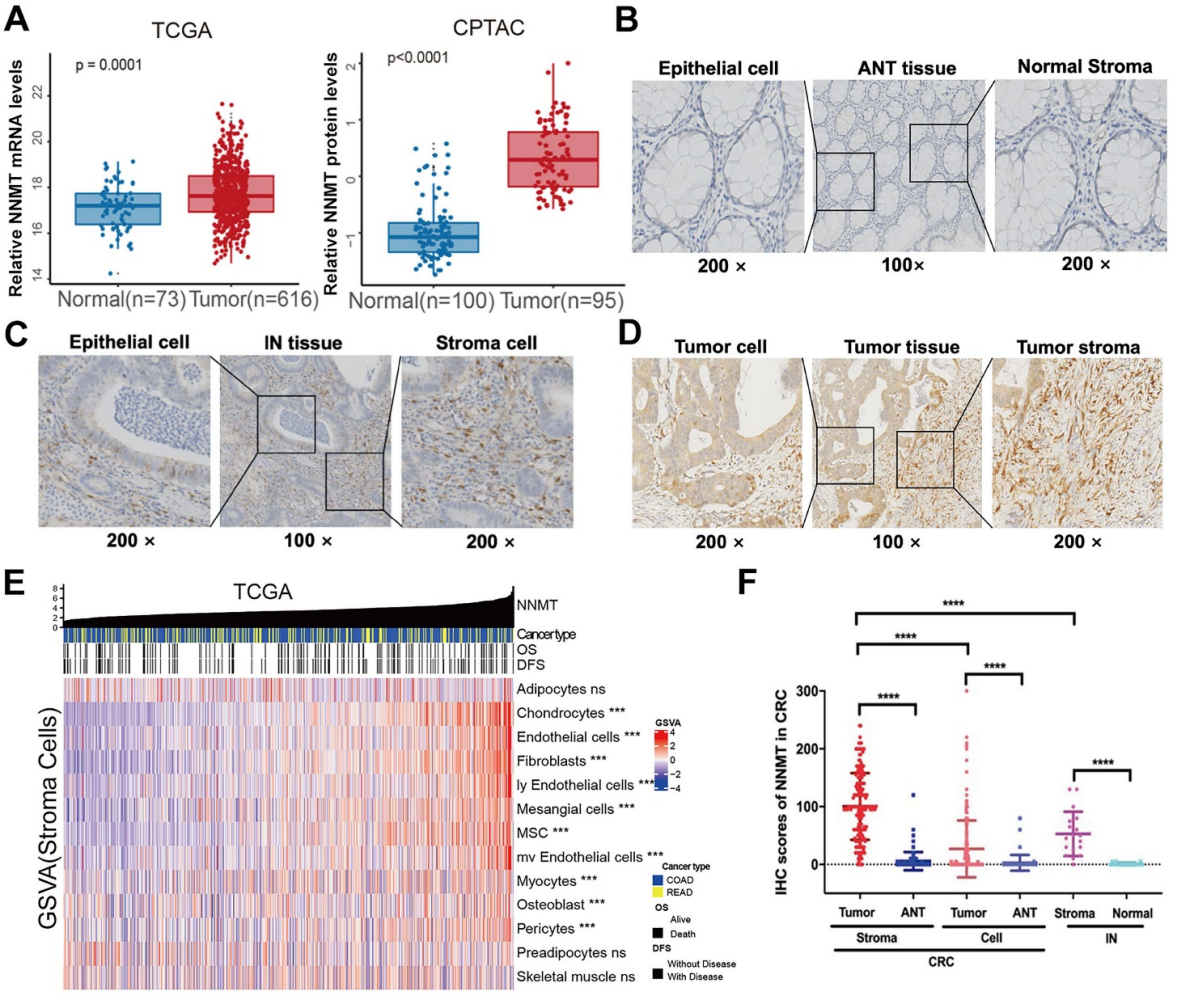
** NNMT is overexpressed in CRC from public datasets and clinical sample IHC result of our study cohort.** (A) Box plots showed higher NNMT expression in tumor tissue compared to ANT in CRC from TCGA and CPTAC datasets. (B). The almost no or extremely low NNMT staining in ANT tissue was observed at low (×100) and high (×200) magnification, respectively. (C) The moderate NNMT staining in IN tissue was observed at low (×100) and high (×200) magnification, respectively. (D) The high NNMT staining in tumor tissue was observed at low (×100) and high (×200) magnification, respectively. (E) The relationship of NNMT expression with stroma component in CRC tissue (TCGA). (F) The quantified results of NNMT expression by IHC in colorectal cancer. (*p<0.05, **p<0.01, ***p<0.001, NS=no signification)

The expression level of NNMT was examined using the publicly available TCGA and proteomics CPTAC database. For TCGA, a public database, we found that tumor mRNA expression NNMT (n=616) was higher than ANT (n=73) (Fig 1A,* p* = 0.0001). Similarly, NNMT protein expression was detected higher in 95 CRC tissue samples than 100 normal tissues by CPTAC database (Fig 1A, *p* < 0.0001). To validate these results, total of 177 patients with CRC and 18 patients with IN were included. The staining of NNMT by IHC was majorly covered the stromal cells characteristically surrounding the epithelial lesions, and also covered some part of tumor cells. None or extremely low NNMT expression was observed in the normal colorectal mucosa (Fig. 1B-D). In addition, by using the current gene sets of stromal cells from the xCell R packages, we generated the expression of stromal cells, such as fibroblasts, endothelial cells, etc., Fig.1E showed that the strong relationship of NNMT with most of stromal cells (*p* < 0.05). Concomitantly, for our results, the score of NNMT expression in tumor cell were significantly higher than that in ANT and IN tissues. Besides, the score of NNMT expression in tumor stroma cells were higher than tumor cells and the score of NNMT expression in tumor stroma were significantly higher than those in stroma of ANT and IN tissues (Fig. 1F, *p* < 0.001). These results indicated that NNMT was highly expressed both in tumor cells and stroma cells of CRC, especially stroma cells.

### The association of NNMT expression with clinicopathologic features and survival in CRC

To further explore the role of NNMT expression in CRC tumor, we analyzed the relationships between NNMT expression in tumor cells or stroma cells and clinicopathologic characteristics of CRC patients in our CRC cohort presented in Table 1 and Table 2. According to the cutoff value (score 20) for distinguishing tumor cell from normal colorectal tissue by NNMT expression in tumor cells, patients were classified into two groups, NNMT^N^ group with score less than 20 and NNMT^P^ group with score more than 20. As shown in Table 1, the positive percentages of NNMT in the tumor cell were 31.6% (56/177). Strikingly, the positive expression of NNMT in tumor cell was significantly correlated with advanced TNM stage (*p*=0.001), lymph node metastasis (*p*=0.001), and distant metastasis (*p*=0.000), while had no significant related with age, gender, and histology (*p* > 0.05, Table 1). In consideration of especially high expression of NNMT in stroma of tumor, patients were classified by the median value (score 100) of NNMT expression in stroma of tumor. Therefore, the score 0-99 was considered as low expression (NNMT^L^), whereas 100-300 as high expression (NNMT^H^). As shown in Table 2, the high percentages of NNMT in the tumor stromal cells were 50.3% (89/177). And, the high expression of NNMT in tumor stromal cells was significantly correlated with advanced TNM stage (*p*=0.026), lymph node metastasis (*p*=0.036), and distant metastasis (*p*=0.050), while had no significant related with age, gender, and histology (*p* > 0.05, Table 2).

**Figure 2:**
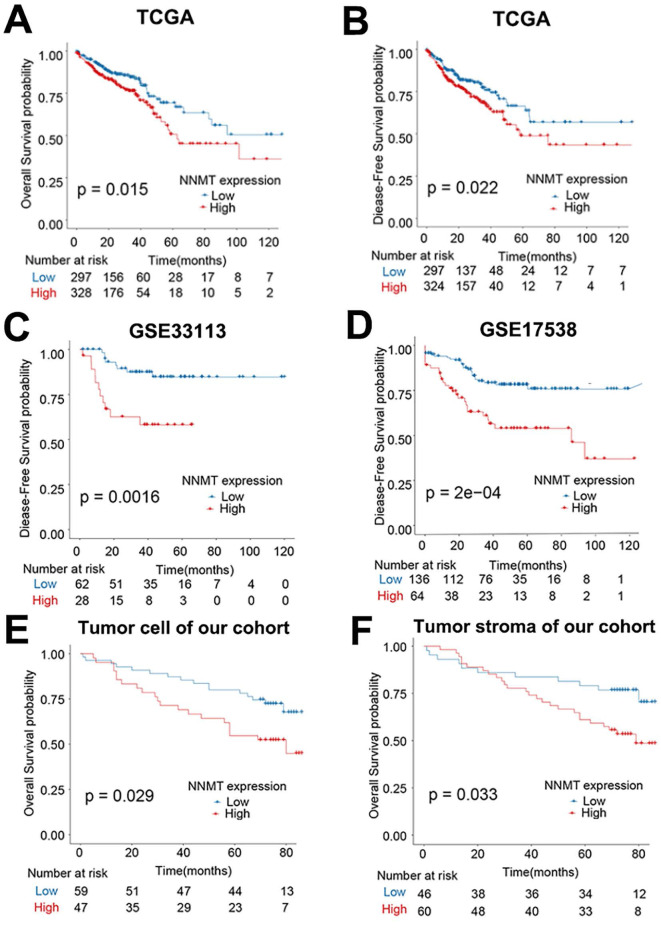
**Overexpression of NNMT predicts short survival in CRC patients.** (A-B) The OS and DFS of CRC patients classified by NNMT expression from TCGA datasets. (C-D) The DFS of CRC patients classified by NNMT expression in GSE33113 and GSE17538 datasets. (E-F) Our 106 clinical CRC patients' survival curves showed that higher expression of NNMT both in tumor cell and stroma indicate poor overall survival.

In addition, in the public TCGA cohort, higher NNMT mRNA expression was significantly related the poor OS and DFS (all *p* < 0.05, Fig. 2A-B). Given that GSE33113 and GSE17538 datasets, higher NNMT mRNA expression also significantly related with poor DFS (all *p* < 0.05, Fig. 2C-D). Similarly, the OS of the patients with positive NNMT expression in tumor cell was significantly shorter than that of patients with low NNMT expression group (*p*=0.029) in our clinical study. The survival of the patients with high NNMT expression in tumor stroma was significantly shorter than that of patients with low NNMT expression (*p*=0.033) (Fig. 2E-F). All of this bioinformatical analysis, which was consistent with our results and strongly indicated that high NNMT expression in CRC is associated with short survival.

### The construction of cell models with NNMT overexpression in SW480 cells and NNMT downregulation in HT-29 cells

To better demonstrate the role of NNMT in metastasis of CRC, we firstly detected the expression of NNMT in five CRC cell lines (DLD1, SW480, SW620, HCT116 and HT-29) by Western blot. HT-29 cells showed high expression of NNMT, while DLD1, SW480, SW620 and HCT116 cells showed no NNMT expression (Fig.S1A). The cell lines HCT116 and SW480 those lack constitutive NNMT expression and HT-29 that has high endogenous NNMT expression were selected as the candidate for further study. Then, SW480 cells were transfected with pcDNA3.1-NNMT vector (SW480/NNMT-1, SW480/NNMT-2) or pcDNA3.1 control vector (SW480/Vector), whereas HT-29 cells were infected with lentiviral NNMT shRNA (HT-29/NNMT shRNA-1, HT-29/NNMT shRNA-2) or lentiviral shRNA NC as a negative control (HT-29/NC). SW480/NNMT-1 and SW480/NNMT-2 cells showed significant higher NNMT expression than SW480/Vector cells, whereas HT-29/NNMT shRNA-1 and HT-29/NNMT shRNA-2 cells showed significant low NNMT expression than HT-29/NC cells, which indicated the cell models were successfully constructed (Fig. S1B).

### NNMT enhances the invasion capacity in CRC cells

**Figure 3:**
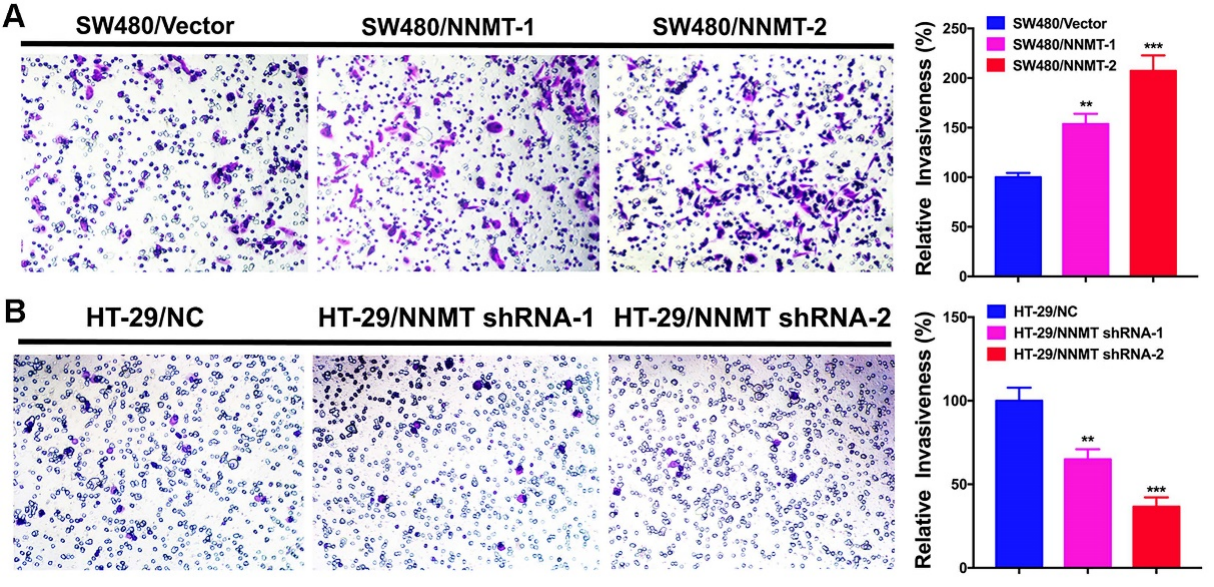
** NNMT promotes the invasion of CRC cells by transwell assay.** (**A**) Overexpression of NNMT in SW480 cells significantly promoted the invasion. (**B**) Knockdown of NNMT in HT-29 cells significantly decreased the invasion. The images are representative results of three independent experiments. The quantitative results were calculated by three independent experiments. (**p<0.01, ***p<0.001)

According to the correlation between NNMT expression and lymph node metastasis and distant metastasis in CRC, we carried out the transwell assay to determine whether NNMT expression affects the cell invasion in CRC cells. As shown in (Fig.3), SW480/NNMT-1 and SW480/NNMT-2 with NNMT overexpression showed the significant larger number of invasion cells than that of SW480/Vector cells, whereas HT-29/NNMT shRNA-1 and HT-29/NNMT shRNA-2 with NNMT downregulation showed the significant smaller number of invasion cells compared with HT-29/NC cells. The relative invasiveness in SW480/NNMT-1 and SW480/NNMT-2 cells were higher than in SW480/Vector cells. Moreover, the relative invasiveness of SW480/NNMT-2 cells is approximately twice as much as SW480/Vector cells. In contract, the relative invasiveness of HT-29/ NNMT shRNA-1 and HT-29/ NNMT shRNA-2 cells were significantly lower than HT-29/NC cells and the relative invasiveness of HT-29/NNMT-2 cells are approximately half of HT-29/NC cells (Fig.3). These results revealed that NNMT promoted the invasion of CRC cells *in vitro*.

### Tumor stromal cells with high NNMT secret 1-MNA to promote cell migration and invasion of CRC cells

**Figure 4:**
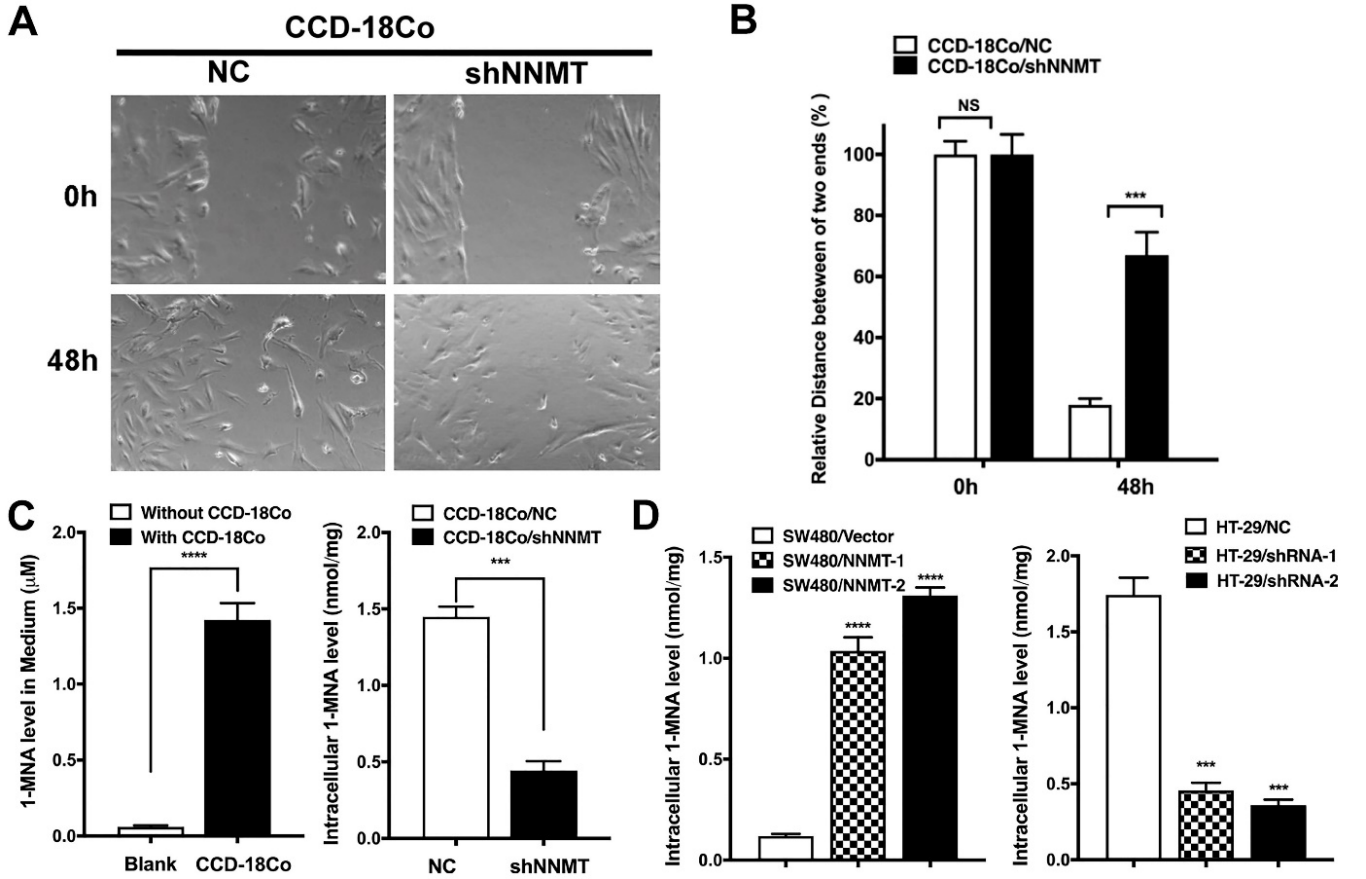
** High NNMT in stromal cells promotes migration by 1-MNA.** (**A, B**) The migration was significantly decreased in CCD-18Co cells after downregulation of NNMT for 48 h by wound healing assay. **(C)** The 1-MNA level in medium with CCD-18Co cell culture was increased, whereas the intracellular 1-MNA of CCD-18Co was decreased after downregulation of NNMT. **(D)** The intracellular 1-MNA level of SW480 cells was increased after overexpression of NNMT, whereas the intracellular 1-MNA of HT-29 cells was decreased after downregulation of NNMT.

Our previous study has reported the effect of NNMT on promotion of cell cycle progression in CRC cells due to the concentration of 1-MNA 23. Therefore, we hypothesized that tumor stromal cells with high NNMT could secrete 1-MNA to promote cell migration and invasion of CRC cells. To confirm this hypothesis, we first purchased the only human colon fibroblast cell line CCD-18Co from ATCC and found CCD-18Co showed high NNMT expression. Second, we found downregulation of NNMT inhibited the migration of CCD-18Co cells, which is consistence with the result of CRC cells (Fig. 4A and B). Third, 1-MNA levels were increased in the medium with CCD-18Co culture and downregulation of NNMT decreased intracellular 1-MNA levels of CCD-18Co cells (Fig. 4C). The same happened in SW480 and HT-29 cell models (Fig. 4D). These results showed that tumor stomal cells with high NNMT increased intracellular 1-MNA levels and secreted 1-MNA, which could promote cell migration and invasion of CRC cells.

**Figure 5:**
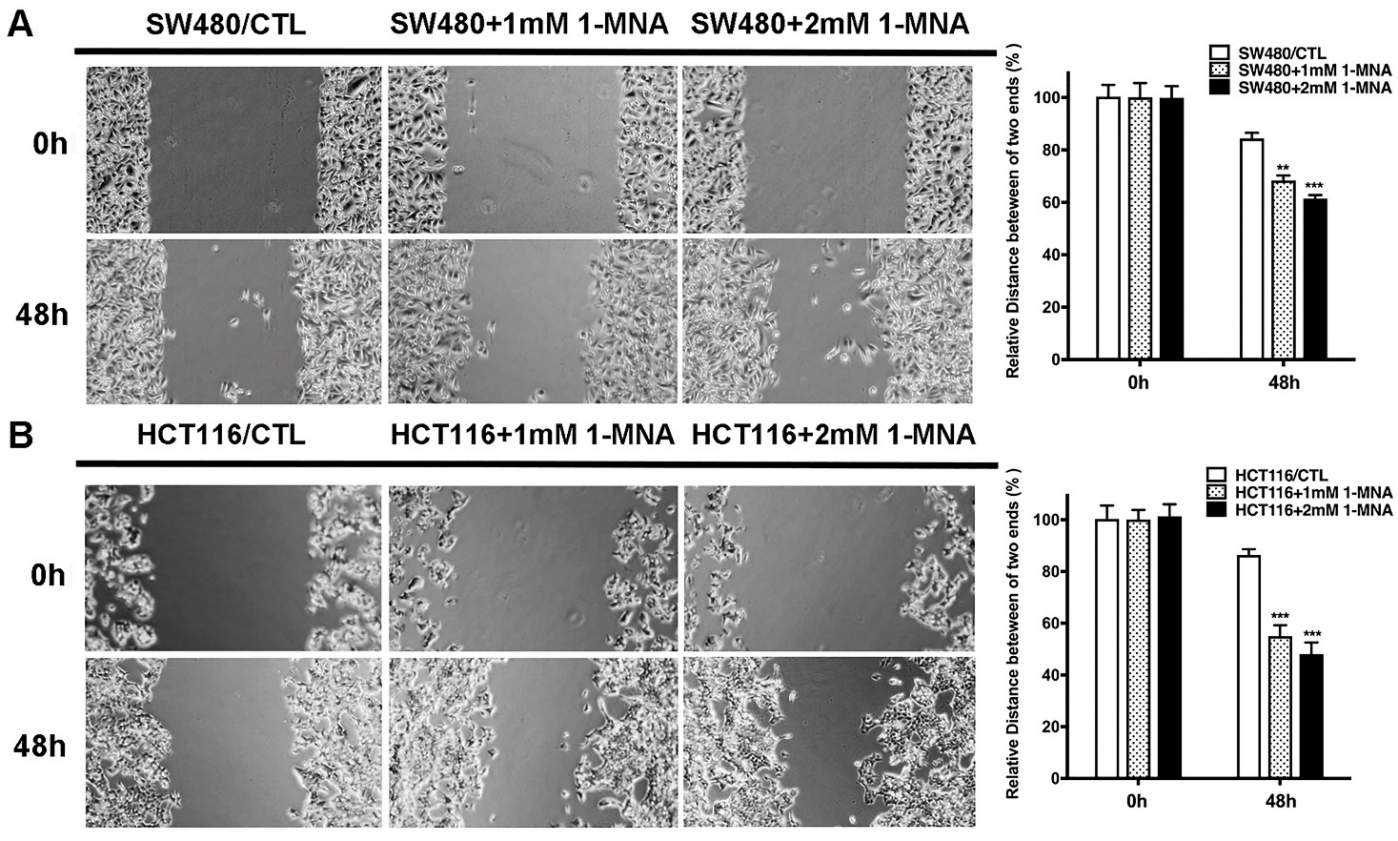
**1-MNA promotes the migration of CRC cells by wound healing assay.** (**A**) The migration was significantly increased in SW480 cells after pre-treatment at a final concentration of 1mM or 2mM 1-MNA for 48 h by wound healing assay. (**B**) The migration was significantly increased in HCT116 cells after pre-treatment at a final concentration of 1mM or 2mM 1-MNA for 48 h by wound healing assay. The images are representative results of three independent experiments. The quantitative results were calculated by three independent experiments. (**p<0.01, ***p<0.001)

**Figure 6:**
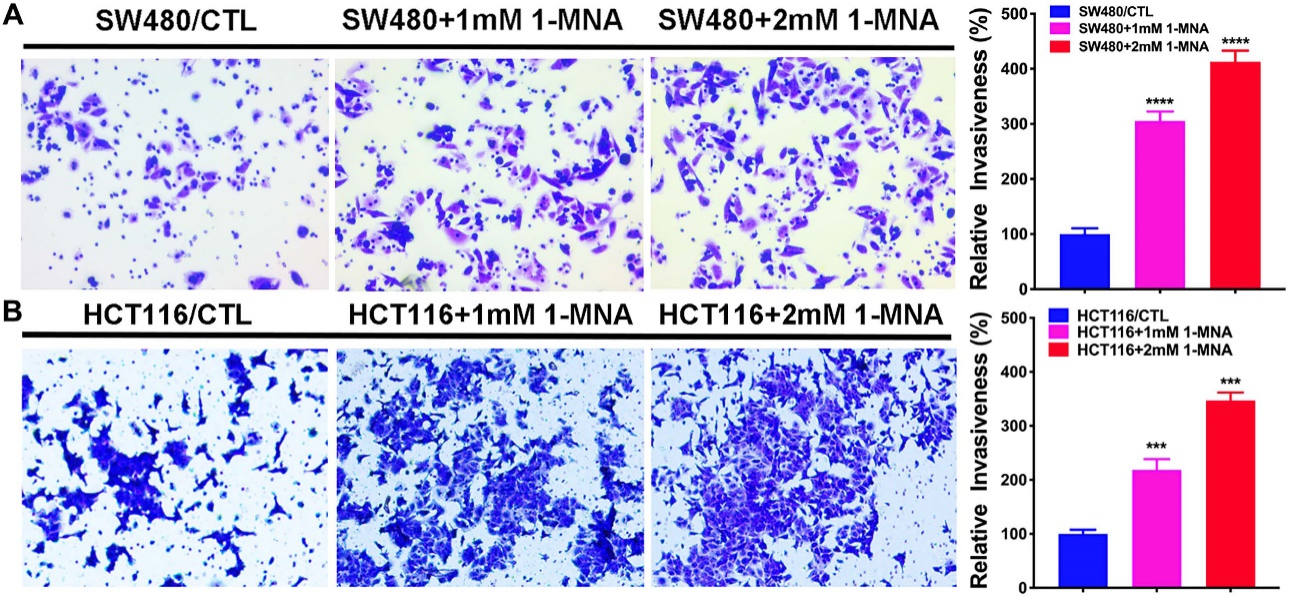
**1-MNA promotes the invasion in CRC cells by transwell assay.** (**A**) The invasion was significantly increased in SW480 cells after pre-treatment at a final concentration of 1mM or 2mM 1-MNA for 48 h by transwell assay. (**B**) The invasion was significantly increased in HCT116 cells after pre-treatment at a final concentration of 1mM or 2mM 1-MNA for 48 h by transwell assay. The images are representative results of three independent experiments. The quantitative results were calculated by three independent experiments. (***p<0.001, ****p<0.0001)

Then, we assessed the effect of 1-MNA on CRC cell migration and invasion instead of NNMT overexpression using wound healing and transwell assays. We found that the SW480 and HCT116 cells with 1-MNA (1mM and 2mM) treatment significantly increased cell migration compared with each control group SW480 and HCT116 cells (Fig.5). The transwell assay also showed that the SW480 and HCT116 cells with 1-MNA incubation exhibited significantly the improved capacity of invasion compared with each control without 1-MNA (Fig. 6).

Taken together, our results demonstrated that tumor stromal cells with high NNMT secret 1-MNA to promote cell migration and invasion of CRC cells* in vitro.*

## Discussion

CRC is one of the most frequently diagnosed gastrointestinal cancers and one of the leading causes of cancer-related deaths worldwide 34. There are several conventional methods for CRC treatment including surgical resection, radiation therapy, chemotherapy, immunotherapy and targeted therapy 35. Despite great advancement in therapeutic approaches, the recurrence and distant metastasis still are major causes of mortality in CRC 36.

Increasing lines of evidence showed that NNMT is significantly overexpressed in various malignancies 8, 9, 11, 15, 18, 19, 21, 22. Interestingly, we showed that NNMT is also highly expressed in CRC, especial in tumor stroma, but is hardly found to be expressed in ANT, which indicated that NNMT is mainly resided in the tumor stroma of CRC. In addition, the moderate elevated expression of NNMT in stroma of IN tissues also indicated that NNMT expression in stroma may be associated with cancerous transformation process. Then, we found that high expression of NNMT both in tumor cell and stroma are associated with advanced TNM stage, lymph node metastasis and distant metastasis, as well as poor OS, which indicated NNMT may play a vital role in metastasis of CRC. Consistence with our results, the recent study utilized laser capture microdissection (LCM) method to confirm that the expression of NNMT in the CAFs is significantly higher than that in tumor cells and leads to ovarian cancer progression by metabolic regulation of histone methylation, which causes transcriptional and epigenetic changes in stromal cells to promote cancer cell migration and metastasis 18.

Furthermore, we found that most CRC cell lines do not have high NNMT expression except HT-29 cells by detecting NNMT expression in five CRC cell lines, which strongly answers the question why the IHC showed NNMT overexpression in few cases of tumor cells, but largely high expression in surrounding stroma. To validate the function of NNMT in metastasis of CRC, we upregulated NNMT expression by a pcDNA3.1/NNMT plasmid into SW480 cells and downregulated NNMT by a shRNA lentiviral vector against NNMT into HT-29 cells, respectively. The overexpression of NNMT significantly promotes the invasion of SW480 cells (Fig. 3A). Conversely, knockdown of NNMT significantly reduces the invasion of HT-29 cells (Fig. 3B). Our results are consistent with the reports derived from squamous cell carcinoma cells 37, esophageal squamous cell carcinoma cells 19, hepatocellular carcinoma cells 21, gastric cancer cells 15, 16, which strongly suggests that the cellular enzyme NNMT serves a crucial role in the migration and invasion of cancer cells.

As we know, 1-MNA is the product of NNMT in the process of catalyzing methyl transfer to nicotinamide, which is significantly positive correlated with NNMT expression 18, 23, 38-40. The previous studies consistently reported that 1-MNA could be secreted into extracellular space and detected in plasma and somehow substitute the biological function of NNMT in cancers 41-43. In our study, we also found the tumor stromal cells with high NNMT increased intracellular 1-MAN and secreted 1-MNA to culture medium. Our previous study has reported the effect of NNMT on promotion of cell cycle progression in CRC cells due to the concentration of 1-MNA 23. This reminder us whether exogenous 1-MNA from tumor stromal cells led to an increased capacity of invasion and migration of CRC cells. To gain an insight into the potential mechanism of 1-MNA on tumor progression, we incubated SW480 and HCT116 cells with certain concentration of 1-MNA to analyze the change of migration and invasion capacity. Our results are reasonable to believe that NNMT enhances the cell invasion and migration capacity via 1-MNA (Fig. 5 and 6), which is similar with the results concluded by Olesya A. Ulanovskaya 39. In addition, both clinical and experimental data suggest that cancer cells gain increased metastatic potential by undergoing EMT. Moreover, previous study showed that cancer cells contacted with CAFs have decreased E-cadherin expression, which reveals that CAFs may regulate the invasion and migration of cancer cells by EMT 44. Combing with our results, the tumor stroma with high NNMT expression may promote migration and invasion of cancer cells in CRC through secreting 1-MNA. Interestingly, it was latest research implied a model whereby tumor and CAFs cells could secrete 1-MNA into the extracellular tumor microenvironment and increased proportion of T cells in ovarian cancer 45, which is consistence with our result.

Our study clearly revealed the feature of NNMT expression in tumor cell and stromal cells in CRC and the mechanism of metastasis. However, our study still has some limitations. Firstly, the larger numbers of cases with long term follow up are necessary to assess the prognostic value of NNMT expression in tumor cells and stromal cells. Second, we did not successfully obtain the CAFs directly from CRC patients to assess the effect of high NNMT expression on migration and invasion of CRC cells by co-culture, which could deepen our study.

## Conclusions

In summary, based on various profile datasets and integrated bioinformatics analysis, we proved that NNMT is highly expressed in CRC tissue than in ANT. Moreover, high NNMT expression in CRC was associated with poor OS and DFS. Our clinical samples revealed that NNMT is highly expressed in tumor of CRC tissue, especially in tumor stroma. The positive expression of NNMT in tumor cell and high expression of NNMT in tumor stroma are correlated with advanced TNM stage, lymph node metastasis, distant metastasis and poor OS. Further functional study verified that the NNMT and its product 1-MNA both play an important role in migration and invasion of CRC cells. Collectively, our serial experiments taken together may open a promising research area of mechanisms underlying the cross-talk between stroma and tumor cell in CRC, which deepens our understanding of tumor progression and offers a new idea for different cancer therapeutic strategies.

## Supplementary Material

Supplementary figure S1.

## Figures and Tables

**Table 1:** The relationship between positive expression of NNMT in tumor cell and clinical pathological characteristics in 177 patients with CRC.

Characteristics	n		Tumor cell	
		NNMT^P^ (%)	Pearsonχ^2^	*P* value
Total	177	56(31.6)		
Gender			0.655	0.418
Male	119	40(33.6)		
Female	58	16(27.6)		
Age			0.14	0.708
<61	88	29(33.0)		
>=61	89	27(30.3)		
TNM			10.573	0.001*
Ⅰ-Ⅱ	104	23(22.1)		
Ⅲ-Ⅳ	73	33(45.2)		
T			1.746	0.186
T_1_+T_2_	21	4(19.0)		
T_3_+T_4_	156	52(33.3)		
Lymph node metastasis			10.607	0.001*
N_0_	107	24(42.9)		
N_1-2_	70	32(57.1)		
Distance metastasis			15.259	0.000*
M_0_	159	43(27.0)		
M_1_	18	13(72.2)		
Histology			3.664	0.300
Mucinous adenocarcinoma	23	5(21.7)		
Non-Mucinous adenocarcinoma	148	49(33.1)		
Squamous cell carcinoma	5	1(20.0)		
Signet-ring cell carcinoma	1	1(100.0)		

NNMT^P^: NNMT positive expression

**Table 2:** The relationship between positive expression of NNMT in tumor stroma and clinical pathological characteristics in 177 patients with CRC.

Characteristics	n		Stroma	
		NNMT^H^ (%)	Pearsonχ^2^	*P* value
Total	177	89(50.3)		
Gender			0.003	0.985
Male	119	60(50.4)		
Female	58	29(50.0)		
Age			0.051	0.821
<61	88	45(51.1)		
>=61	89	44(49.4)		
TNM			4.961	0.026*
Ⅰ-Ⅱ	104	45(43.3)		
Ⅲ-Ⅳ	73	44(60.3)		
T			1.287	0.257
T_1_+T_2_	21	13(61.9)		
T_3_+T_4_	156	76(48.7)		
Lymph node metastasis			4.374	0.036*
N_0_	107	47(43.9)		
N_1-2_	70	42(60.0)		
Distance metastasis			3.858	0.050*
M_0_	159	76(47.8)		
M_1_	18	13(72.2)		
Histology			4.557	0.207
Mucinous adenocarcinoma	23	9(39.1)		
Non-Mucinous adenocarcinoma	148	79(53.4)		
Squamous cell carcinoma	5	1(20.0)		
Signet-ring cell carcinoma	1	0(0.0)		

NNMT^H^: NNMT high expression

## Abbreviations

1-MNA: 1-methyl-nicotinamide
ANT: adjacent normal tissue
CAFs: cancer-associated fibroblasts
COAD: colon cancer
CPTAC: Clinical Proteomic Tumor Analysis Consortium
CRC: colorectal cancer
DAB: diaminobenzidine
DFS: Disease-free survival
ECM: extracellular matrix
EMT: epithelial-mesenchymal transition
GEO: Gene Expression Omnibus
IN: intraepithelial neoplastic
IHC: immunohistochemistry
LCM: laser capture microdissection
NA: nicotinamide
NNMT: Nicotinamide N-methyltransferase
OS: overall survival
READ: rectal cancer
SAM: *S*-5′-adenosyl-L-methionine
TCGA: The Cancer Genome Atlas
TNM: tumor-node-metastasis
TMA: tissue microarray
WHO: World Health Organization

## References

[1] Siegel R L, Miller K D, Goding Sauer A (2020). Colorectal cancer statistics, 2020. CA Cancer J Clin.

[2] Zhu J, Tan Z, Hollis-Hansen K (2017). Epidemiological Trends in Colorectal Cancer in China: An Ecological Study. Dig Dis Sci.

[3] Chen W (2015). Cancer statistics: updated cancer burden in China. Chin J Cancer Res.

[4] Sun ZQ, Ma S, Zhou QB (2017). Prognostic value of lymph node metastasis in patients with T1-stage colorectal cancer from multiple centers in China. World J Gastroenterol.

[5] Takahashi H, Berber E (2020). Role of thermal ablation in the management of colorectal liver metastasis. Hepatobiliary Surg Nutr.

[6] Hong S, Moreno-Navarrete J M, Wei X (2015). Nicotinamide N-methyltransferase regulates hepatic nutrient metabolism through Sirt1 protein stabilization. Nat Med.

[7] Sternak M, Jakubowski A, Czarnowska E (2015). Differential involvement of IL-6 in the early and late phase of 1-methylnicotinamide (MNA) release in Concanavalin A-induced hepatitis. Int Immunopharmacol.

[8] Zhang J, Xie X Y, Yang S W (2010). Nicotinamide N-methyltransferase protein expression in renal cell cancer. J Zhejiang Univ Sci B.

[9] Wang Y, Zeng J, Wu W (2019). Nicotinamide N-methyltransferase enhances chemoresistance in breast cancer through SIRT1 protein stabilization. Breast Cancer Res.

[10] Zhang J, Wang Y, Li G (2014). Down-regulation of nicotinamide N-methyltransferase induces apoptosis in human breast cancer cells via the mitochondria-mediated pathway. PLoS One.

[11] Tomida M, Mikami I, Takeuchi S (2009). Serum levels of nicotinamide N-methyltransferase in patients with lung cancer. J Cancer Res Clin Oncol.

[12] Bach D H, Kim D, Bae S Y (2018). Targeting Nicotinamide N-Methyltransferase and miR-449a in EGFR-TKI-Resistant Non-Small-Cell Lung Cancer Cells. Mol Ther Nucleic Acids.

[13] Sartini D, Muzzonigro G, Milanese G (2013). Upregulation of tissue and urinary nicotinamide N-methyltransferase in bladder cancer: potential for the development of a urine-based diagnostic test. Cell Biochem Biophys.

[14] Akar S, Harmankaya I, Ugras S (2020). Expression and Clinical Significance of Nicotinamide N-Methyltransferase in Cervical Squamous Cell Carcinoma. Int J Gynecol Pathol.

[15] Chen C, Wang X, Huang X (2016). Nicotinamide N-methyltransferase: a potential biomarker for worse prognosis in gastric carcinoma. Am J Cancer Res.

[16] Liang L, Zeng M, Pan H (2018). Nicotinamide N-methyltransferase promotes epithelial-mesenchymal transition in gastric cancer cells by activating transforming growth factor-beta1 expression. Oncol Lett.

[17] Kanska J, Aspuria PP, Taylor-Harding B (2017). Glucose deprivation elicits phenotypic plasticity via ZEB1-mediated expression of NNMT. Oncotarget.

[18] Eckert M A, Coscia F, Chryplewicz A (2019). Proteomics reveals NNMT as a master metabolic regulator of cancer-associated fibroblasts. Nature.

[19] Cui Y, Zhang L, Wang W (2019). Downregulation of nicotinamide N-methyltransferase inhibits migration and epithelial-mesenchymal transition of esophageal squamous cell carcinoma via Wnt/beta-catenin pathway. Mol Cell Biochem.

[20] Kim J, Hong S J, Lim E K (2009). Expression of nicotinamide N-methyltransferase in hepatocellular carcinoma is associated with poor prognosis. J Exp Clin Cancer Res.

[21] Li J, You S, Zhang S (2019). Elevated N-methyltransferase expression induced by hepatic stellate cells contributes to the metastasis of hepatocellular carcinoma via regulation of the CD44v3 isoform. Mol Oncol.

[22] Akar S, Harmankaya I, Ugras S (2019). Nicotinamide N-methyltransferase expression and its association with phospho-Akt, p53 expression, and survival in high-grade endometrial cancer. Turk J Med Sci.

[23] Xie X, Yu H, Wang Y (2014). Nicotinamide N-methyltransferase enhances the capacity of tumorigenesis associated with the promotion of cell cycle progression in human colorectal cancer cells. Archives of Biochemistry and Biophysics.

[24] Song M, Li Y, Miao M (2020). High stromal nicotinamide N-methyltransferase (NNMT) indicates poor prognosis in colorectal cancer. Cancer Medicine.

[25] Zhang L, Song M, Zhang F (2021). Accumulation of Nicotinamide N-Methyltransferase (NNMT) in Cancer-associated Fibroblasts: A Potential Prognostic and Predictive Biomarker for Gastric Carcinoma. Journal of Histochemistry & Cytochemistry.

[26] Gascard P, Tlsty TD (2016). Carcinoma-associated fibroblasts: orchestrating the composition of malignancy. Genes Dev.

[27] Kalluri R, Zeisberg M (2006). Fibroblasts in cancer. Nature Reviews Cancer.

[28] Ostman A, Augsten M (2009). Cancer-associated fibroblasts and tumor growth-bystanders turning into key players. Curr Opin Genet Dev.

[29] Pietras K, Ostman A (2010). Hallmarks of cancer: interactions with the tumor stroma. Exp Cell Res.

[30] Kobayashi H, Enomoto A, Woods S L (2019). Cancer-associated fibroblasts in gastrointestinal cancer. Nat Rev Gastroenterol Hepatol.

[31] Quail D F, Joyce J A (2013). Microenvironmental regulation of tumor progression and metastasis. Nat Med.

[32] Joyce J A, Pollard J W (2009). Microenvironmental regulation of metastasis. Nat Rev Cancer.

[33] Wang M, Zhao J, Zhang L (2017). Role of tumor microenvironment in tumorigenesis. Journal of Cancer.

[34] Torre L A, Bray F, Siegel R L (2015). Global cancer statistics, 2012. CA Cancer J Clin.

[35] Mishra J, Drummond J, Quazi S H (2013). Prospective of colon cancer treatments and scope for combinatorial approach to enhanced cancer cell apoptosis. Crit Rev Oncol Hematol.

[36] Schirrmacher V, Fournier P, Schlag P (2014). Autologous tumor cell vaccines for post-operative active-specific immunotherapy of colorectal carcinoma: long-term patient survival and mechanism of function. Expert Rev Vaccines.

[37] Hah Y S, Cho H Y, Jo S Y (2019). Nicotinamide Nmethyltransferase induces the proliferation and invasion of squamous cell carcinoma cells. Oncol Rep.

[38] Xie X, Liu H, Wang Y (2016). Nicotinamide N-methyltransferase enhances resistance to 5-fluorouracil in colorectal cancer cells through inhibition of the ASK1-p38 MAPK pathway. Oncotarget.

[39] Ulanovskaya O A, Zuhl A M, Cravatt B F (2013). NNMT promotes epigenetic remodeling in cancer by creating a metabolic methylation sink. Nature Chemical Biology.

[40] Gao Y, van Haren M J, Moret E E (2019). Bisubstrate Inhibitors of Nicotinamide N-Methyltransferase (NNMT) with Enhanced Activity. J Med Chem.

[41] Lu XM, Long H (2018). Neoplasma LHJ. Nicotinamide N-methyltransferase as a potential marker for cancer.

[42] Babault N, Allali-Hassani A, Li F (2018). Discovery of Bisubstrate Inhibitors of Nicotinamide N-Methyltransferase (NNMT). Journal of Medicinal Chemistry.

[43] Kannt A, Rajagopal S, Kadnur S V (2018). A small molecule inhibitor of Nicotinamide N-methyltransferase for the treatment of metabolic disorders. Sci Rep.

[44] Vered M, Dayan D, Yahalom R (2010). Cancer-associated fibroblasts and epithelial-mesenchymal transition in metastatic oral tongue squamous cell carcinoma. International Journal of Cancer.

[45] Kilgour M, MacPherson S, Zacharias L (2021). 1-Methylnicotinamide is an immune regulatory metabolite in human ovarian cancer. Sci Adv.

