# Supplementary material for: Overexpression of Nicotinamide N-methyltransferase mainly covers stroma of colorectal cancer and correlates with unfavorable survival by its product 1-MNA
Source: J Cancer. 2021 Aug 26;12(20):6170–81. doi: 10.7150/jca.56419 (PMC8425209; doi:10.7150/jca.56419)
Supplement: Supplementary file 1 — Supplementary figure S1. [file jcav12p6170s1.pdf]

Supplement Figure S1

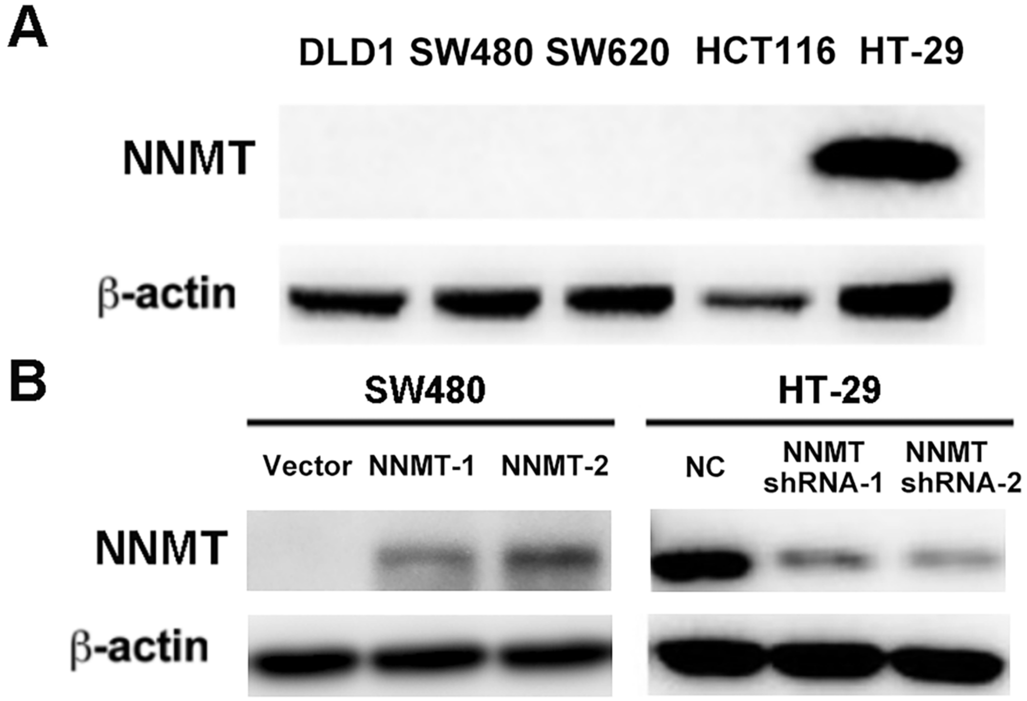

Figure S1. Expression of NNMT in different CRC cell lines and the cell models of NNMT overexpression and downregulation. (**A**) Western blot was used to analyze the NNMT protein expression levels in five CRC cell lines. β-actin was used as the internal control. High protein expression level of NNMT was detected in HT-29, while DLD1, SW480, SW620 and HCT116 cells showed no NNMT expression. (**B**) Western blot was used to analyze the NNMT expression in SW480 cell model with NNMT overexpression and HT-29 cell model with NNMT downregulation. These are the representative results of at least three independent experiments. β-actin was used as an internal control.
